# Natural cytotoxicity receptor splice variants orchestrate the distinct functions of human natural killer cell subtypes

**DOI:** 10.1038/ncomms10183

**Published:** 2015-12-15

**Authors:** Johan Siewiera, Jordi Gouilly, Hocine-Rachid Hocine, Géraldine Cartron, Claude Levy, Reem Al-Daccak, Nabila Jabrane-Ferrat

**Affiliations:** 1 INSERM UMR 1043, Centre de Physiopathologie de Toulouse Purpan (CPTP), Toulouse F-31300, France; 2 CNRS UMR 5282, Toulouse F-31300, France; 3 Université Toulouse III Paul Sabatier, CHU Purpan BP 3028, Toulouse F-31300, France; 4 INSERM UMRS976 and Université Paris-Diderot, Hôpital Saint Louis, Paris 75010, France; 5 Service de Gynécologie-Obstétrique, Centre Hospitalo-Universitaire de Toulouse, Hôpital Paule de Viguier, Toulouse 31059, France; 6 Service de Gynécologie-Obstétrique Clinique Sarrus-Teinturiers, Toulouse 31300, France

## Abstract

The natural cytotoxicity receptors NKp46/NCR1, NKp44/NCR2 and NKp30/NCR3 are critical for natural killer (NK) cell functions. Their genes are transcribed into several splice variants whose physiological relevance is not yet fully understood. Here we report that decidua basalis NK (dNK) cells of the pregnant uterine mucosa and peripheral blood NK (pNK) cells, two functionally distinct subsets of the physiological NK cell pool, display differential expression of NKp30/NCR3 and NKp44/NCR2 splice variants. The presence of cytokines that are enriched within the decidual microenvironment is sufficient to convert the splice variant profile of pNK cells into one similar to that of dNK cells. This switch is associated with decreased cytotoxic function and major adaptations to the secretome, hallmarks of the decidual phenotype. Thus, NKp30/NCR3 and NKp44/NCR2 splice variants delineate functionally distinct NK cell subsets. To our knowledge, this is the first conclusive evidence underlining the physiological importance of NCR splice variants.

## Abstract (web summary)

Decidual natural killer (NK) cells from the pregnant uterus play an important role in the physiology of pregnancy and differ functionally from peripheral blood NK cells. Siewiera *et al*. reveal that this is partly due to the differential expression of splice variants of natural cytotoxicity receptors by these two cell subsets.

## Graphical abstract

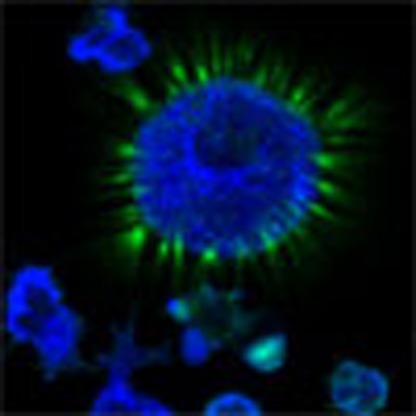

Natural killer (NK) cells are a pool of innate immune cells that play a key role in controlling pathological situations such as viral infections and tumours, as well as more physiological ones such as pregnancy. NK cell effector functions are orchestrated by a wide array of germline-encoded receptors (NKRs) that are expressed in a stochastic pattern. Natural cytotoxicity receptors (NCRs) are among the major activating NKRs that recognize as yet unidentified self-ligands^1^,^2^,^3^. NCRs belong to the immunoglobulin-like family and are involved in NK cell cytotoxic function against infected cells and tumours^4^,^5^,^6^,^7^. The NCRs NKp46/NCR1 and NKp30/NCR3 are expressed on resting and upregulated on activated NK cells, whereas NKp44/NCR2 is expressed only on activated cells. *NCR* genes can be transcribed into several splice variants^4^,^8^. *NCR3* can be transcribed into six different splice variants, with NKp30a, NKp30b and NKp30c being the most abundant isoforms each with distinct functions^9^. NKp30a and NKp30b convey stimulatory signals, whereas NKp30c is immunosuppressive^10^. *NCR1* and *NCR2* also encode several splice variants^4^,^8^, but whereas no functional differences have been observed for the *NCR1* splice variants, studies on *NCR2* have suggested the presence of an inhibitory isoform^11^. Although these studies have shed light on the various NCR alternative splice variants, the physiological and biological relevance of the various NCR isoforms is far from being fully resolved.

Peripheral blood NK (pNK) cells and decidua basalis NK (dNK) cells from the pregnant uterus lining are two distinct subsets of the physiological NK cell pool. They are clearly different at both phenotypic and functional levels^12^,^13^,^14^,^15^,^16^,^17^. In contrast to pNK cells that are CD56^dim^CD16^pos^, most of dNK cells are CD56^bright^CD16^neg^. Furthermore, dNK cells express a unique repertoire of activating and inhibitory NKRs. Although resting pNK cells do not express the NKp44 receptor, dNK cells express all three NCRs. Very little is known about the origin of dNK cells. It is thought that they could derive from NK cell progenitors and/or mature pNK cells that migrate/proliferate/differentiate in a local environment enriched in steroids and cytokines/chemokines^18^,^19^,^20^,^21^,^22^,^23^. Mouse studies have revealed a unique functional role of dNK cells in supporting the implantation of the embryo^24^,^25^. The dNK cells recruited to the early implantation site of mouse decidua basalis secrete numerous factors that contribute to both neo-angiogenesis of early decidual vessels and alterations to the structural components of newly developing and existing vessels. Likewise, human dNK cells actively participate in neo-angiogenesis and contribute towards fetal trophoblast differentiation/invasion and vascular remodelling^14^,^26^,^27^. Similar to pNK cells, dNK cells are also involved in the immune response against various threats^16^.

The molecular basis underlying the differential functions of these two NK cell subsets has not been fully elucidated. Studies on the function of the NCRs suggest that they might each play discreet roles. Although all three NCRs have been shown to induce pNK cell cytotoxicity and cytokine production, only NKp46/NCR1 was able to induce dNK cell cytotoxicity^13^. Moreover, NKp30/NCR3 was shown to promote cytokine secretion, whereas NKp44/NCR2 was found to have an inhibitory function in dNK cells^13^,^14^,^16^. Herein, we examined whether the expression of alternatively spliced variants of the NCRs might delineate these two NK cell subsets and explain their differential function. Analysis of a cohort of dNK and pNK cells from the same donors demonstrates that first-trimester dNK cells express NCR isoforms that are different from those expressed by pNK cells. We find that this differential expression might be physiologically relevant. It considerably has an impact on the lytic activity of dNK cells and is sculpted by cytokines enriched within the decidual microenvironment (decidua-enriched cytokines) that select for the expression of inhibitory rather than activating isoforms of NKp30/NCR3 and NKp44/NCR2. To our knowledge, this is the first study proposing a biological relevance to the various NCR isoforms, whose expression defines the pNK and dNK cell subsets at the molecular level, as well as their differential functions.

## Results

### NCR variants control NK cell subsets degranulation

NCR engagement triggers different effector functions in dNK cells compared with pNK cells^13^. We investigated whether alternatively spliced variants of NCR might underpin the differences between these two NK cell subsets in a cohort of dNK and pNK cells from the same donors. We performed *in silico* multiple sequence alignment of the NKp30/NCR3 and NKp44/NCR2 isoform coding sequences, then designed specific primers to depict the three major isoforms of each receptor and verified their specificity by reverse-transcription PCR (RT–PCR) (Supplementary Fig. 1 and Table 1).

**Figure 1:**
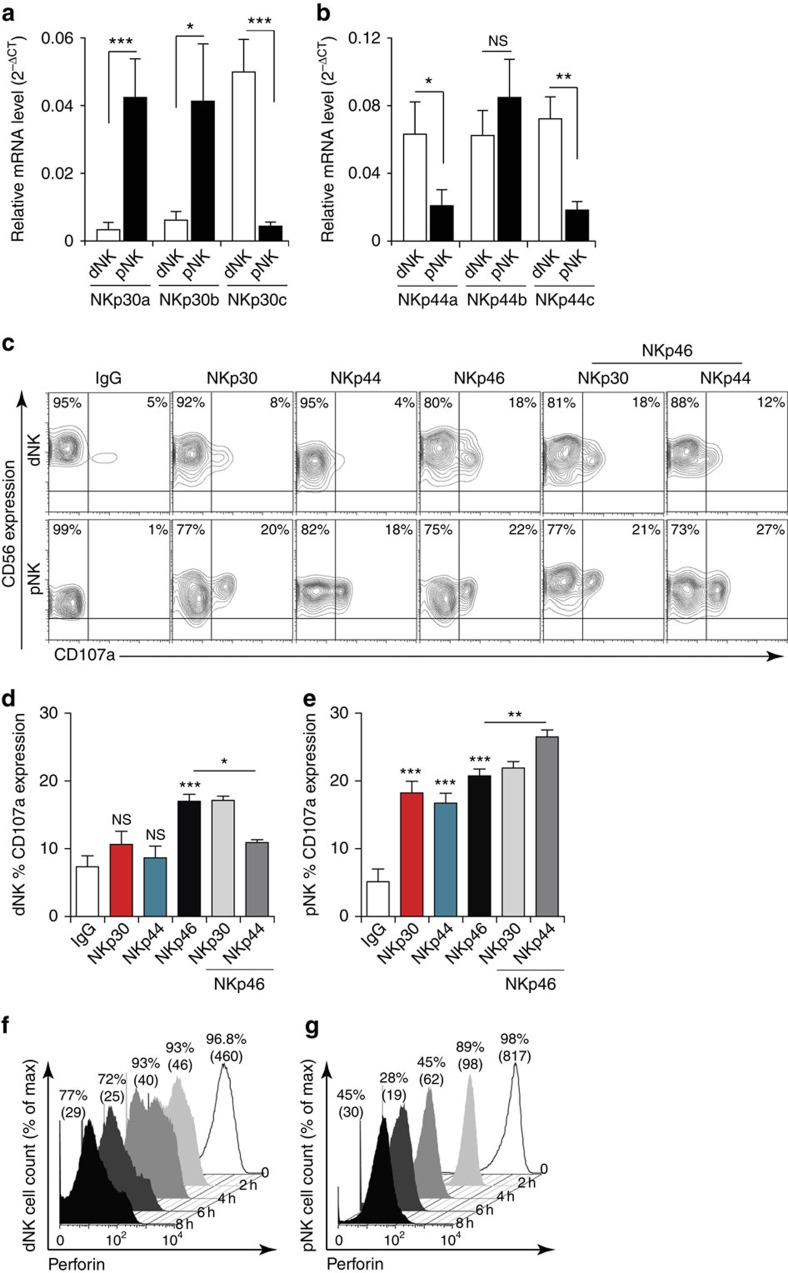
Differential expression of NCR isoforms by dNK and pNK cells results in differential activation on receptor cross-linking. (**a**,**b**) mRNA was extracted from freshly isolated dNK and pNK cells from the same donor (ten matched donor samples). Quantitative reverse-transcription PCR was used to measure the relative expression of the three NKp30/NCR3 (**a**) and NKp44/NCR2 (**b**) splice variants. Data are representative of ten independent donors. Bar graphs show mean values±s.e.m. *P<0.05 and **P<0.01; NS, not significant; Student's *t*-test. (**c**,**d**,**e**) Freshly isolated dNK and pNK cells cultured overnight with IL15 (10 ng ml^−1^) were stimulated for 4 h with a single or combination of two specific monoclonal antibodies, used as ligands. (**c**) NK cell degranulation was assessed by quantification of CD107a cell surface expression using flow cytometry on CD3^neg^CD56^pos^ cells. (**d**) Percentage of freshly isolated dNK cells and (**e**) pNK cells showing CD107a surface expression. Results are presented as mean values±s.e.m. from four independent donors. *P<0.05, **P<0.01, ***P<0.001; NS, not significant; one-way analysis of variance with Bonferroni post test. (**f**,**g**) Representative experiment showing the kinetics of intracellular perforin expression monitored for 8 h in the presence of polyclonal stimulation with ionomycin/phorbol myristate acetate (PMA; *n*=4). The percentage of NK cells expressing perforin is given above each FACS histogram; MFI values are shown in brackets.

Consistent with both a previous report^10^ and our RT–PCR results, pNK cells predominantly expressed NKp30a and NKp30b messenger RNA but almost no NKp30c mRNA (Fig. 1a). In contrast, dNK cells expressed significantly lower amounts of NKp30a and NKp30b mRNA but very high levels of NKp30c mRNA (Fig. 1a). Compared with pNK cells, dNK cells expressed at least tenfold more NKp30c. On the other hand, dNK cells displayed similar expression levels of all three NKp44 mRNAs, whereas pNK cells exclusively expressed NKp44b mRNA (Fig. 1b). The relative ratios further demonstrated that freshly isolated pNK cells expressed NKp44b mRNA at levels that were greater than NKp44a and NKp44c mRNA. Levels of NKp44a and NKp44c mRNA in dNK cells were at least three and four times higher than in pNK cells, respectively.

To examine the relevance of the observed differences in NCR isoform expression profiles in dNK versus pNK cells, we investigated their impact on NK cell effector functions. We monitored the cellular degranulation of dNK and pNK cells on NCR ligation as readout of lytic activity. As freshly isolated pNK cells, in contrast to dNK cells, lack the cell surface expression of NKp44, therefore they were cultured overnight with 10 ng ml^−1^ of interleukin-15 (IL15). These CD3^neg^CD56^pos^ pNK and dNK cells were activated for 4 h with specific anti-NCR antibodies, then analysed for cell surface expression of the CD107a degranulation marker. Ligation of the NKp30, NKp44 or the IgG isotype-matched control on dNK cells did not increase the CD107a expression, whereas ligation of NKp46 induced degranulation in 18% of dNK cells (Fig. 1c). Co-engagement of the NKp44 and NKp46 receptors decreased NKp46-induced degranulation by >30% in dNK cells, whereas co-ligation of NKp30 had no effect (Fig. 1c,d). In line with previous reports^28^,^29^,^30^, NKp30, NKp44 and NKp46 ligation all resulted in a significant increase in CD107a expression in pNK cells (Fig. 1c,e). Simultaneous ligation of NKp44 and NKp46 showed an incremental effect on the ability of pNK cells to degranulate, whereas co-engagement of NKp30 and NKp46 had no impact on NKp46-induced degranulation (Fig. 1c,e).

For a complete assessment, we also monitored NK cell degranulation by adding the anti-CD107a antibody and the intracellular transport blocker monensin during the whole stimulation period. The degranulation profiles of pNK cells were similar to those observed when anti-CD107a antibody was added at the end of the degranulation assay. The cumulative increase in CD107a over time was much more pronounced for pNK than for dNK cells (Supplementary Fig. 2a). However, as the baseline level of degranulation was much higher for dNK cells we cannot exclude an eventual side effect of the intracellular transport blocker on dNK cells.

To ensure that the difference observed between dNK and pNK cell was not due to defective dNK cell degranulation, we stimulated the cells with phorbol myristate acetate/ionomycin and monitored their perforin contents over time. In agreement with previous reports^27^,^31^, even though dNK expressed less perforin than pNK cells at the baseline level, 100% of cells of both subsets expressed perforin and were similarly able to degranulate on optimal stimulation (Fig. 1f,g).

Together, these data demonstrate that the differential expression of NKp30 and NKp44 splice variants by dNK and pNK cells delineates their differential ability to release lytic granules on specific receptor engagement. Thus, the molecular individualization of dNK and pNK cells might be of functional and/or biological relevance.

### NCRs splice variants affect NK-cell immune synapse formation

NK cell functions are governed by signalling pathways activated on their receptors ligation and consequent immune synapse (IS) formation. To validate the relevance of the differences in the dNK and pNK subsets, we first analysed some of intracellular signalling events downstream of the NCRs. Ligation of NKp46 on dNK and pNK cells led to fairly similar tyrosine phosphorylation patterns (Supplementary Fig. 2b and Supplementary Methods). However, ligation of NKp30 and NKp44 induced differential tyrosine phosphorylation patterns in the two cell types. We also analysed the phosphorylation of Vav1 and mitogen-activated protein kinase/Erk. Again NKp46 induced fairly similar Vav1 phosphorylation in both cell subsets. NKp30 induced Vav1 phosphorylation mainly in pNK cells, whereas NKp44 induced its phosphorylation mainly in dNK cells. Activation of the mitogen-activated protein kinase/Erk pathway was less clear. NKp30 and NKp46, but not NKp44, appeared to activate Erk1/2 phosphorylation in pNK but not in dNK cells (Supplementary Fig. 2b). Although warranted further investigation, these results suggest that the differential expression of NKp30 and NKp44 isoforms by dNK and pNK cells could lead to differential activation of intracellular signalling on specific ligation of these NCRs.

**Figure 2:**
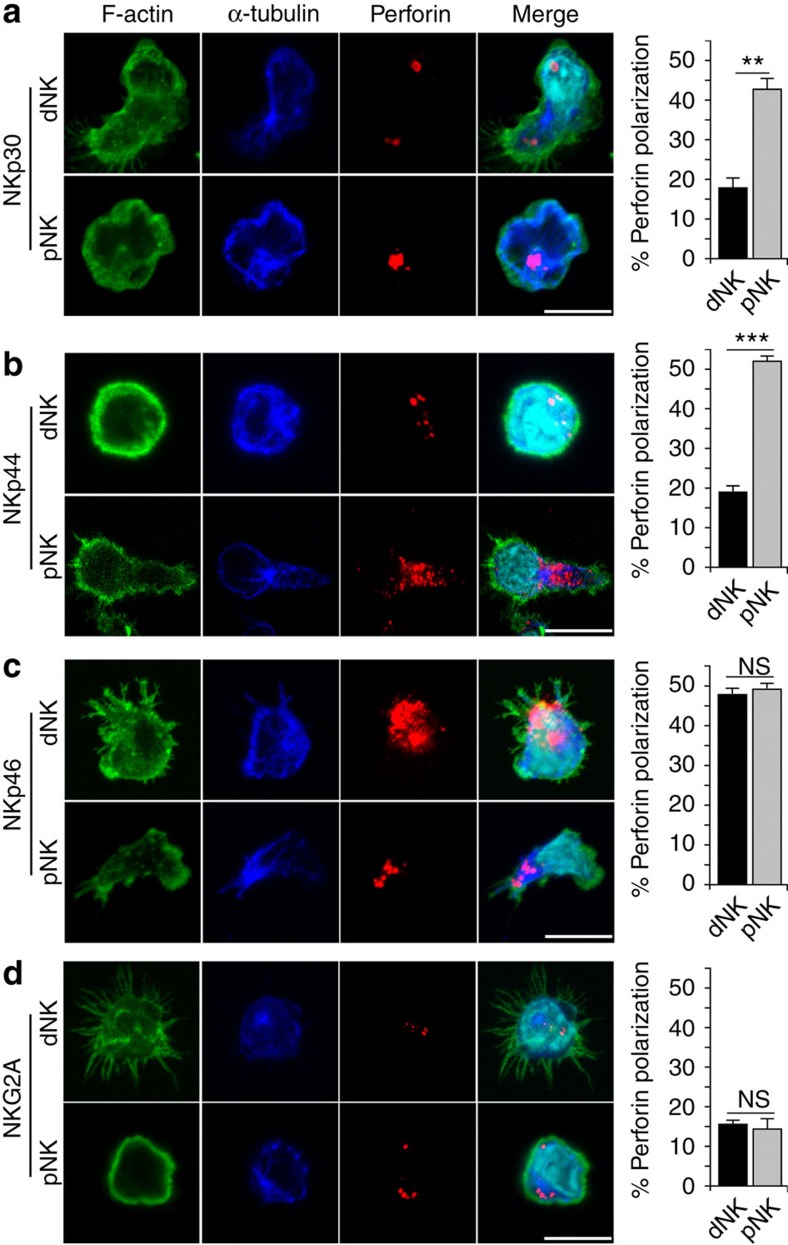
Specific ligation of NCRs leads to differential IS formation. (**a**–**d**) Representative images of IS formation on specific engagement of NCRs of dNK or pNK cells cultured overnight with IL15 stimulated on glass coverslip surfaces coated with anti-NKp30 (**a**), anti-NKp44 (**b**), anti-NKp46 (**c**) and anti-NKG2A (**d**) antibodies. Representative images of NK cells and antibody-coated glass coverslips were acquired with a Zeiss LSM710 confocal microscope using a × 63 oil objective (Carl Zeiss AG, Jena, Germany). Polarization was imaged after 20 min of stimulation. F-actin (phalloidin in green), microtubules (α-tubulin in blue) and lytic granules containing perforin (red) were stained. Scale bar, 20 μm. Images were processed using ImageJ software. Bar graphs (right panel) represent the percentage of conjugates showing polarized perforin after receptor engagement. At least 300 conjugates were analysed. Results are given as average mean values±s.e.m. from five independent experiments. **P<0.01; NS, not significant; Student's *t*-test.

We then examined the organization of ISs after ligation of a single NCR. IS formation is usually associated with cortical actin remodelling. The organization of actin-containing micro-domains at the vicinity of the microtubule organizing centre (MTOC) serves as a docking site for lytic granules^32^. NKp30 ligation on cells from the two NK cell subsets resulted in the rapid reorganization of F-actin-enriched cytoskeleton and the polarization of the MTOC. By counting cells showing a polarized MTOC and perforin-containing granules, we found that >45% of pNK cells displayed polarized IS (Fig. 2a). In contrast, <20% of dNK cells displayed polarized lytic granules after NKp30 ligation. Similar to NKp30, NKp44 ligation induced organized IS in >50% of pNK cells, whereas only minor effects were seen in dNK cells (Fig. 2b). In agreement with the degranulation assay, activation through NKp46 ligation had similar effects in pNK and dNK cells with almost 50% of cells sharing features of cytolytic IS (Fig. 2c). As a negative control, <20% of cells exhibited organized IS after ligation of the NKG2A inhibitory receptor (Fig. 2d).

Overall, and in line with the degranulation activities, all three NCRs induced pNK cell lytic IS formation, whereas only NKp46 ligation triggered lytic IS in dNK cells. This further supports that the differential expression of NCR splice variants by dNK and pNK cells could contribute to their distinct functioning.

### Decidua-enriched cytokines modulate NCR isoform expression

The above findings prompted us to investigate the elements that might contribute towards sculpting the expression profile of NCR splice variants in NK cell subsets. dNK and pNK cells operate within distinct microenvironments. The decidual stroma produces transforming growth factor-β (TGF-β) and the pro-inflammatory IL15 and IL18, which are critical for healthy pregnancy^33^,^34^,^35^.

**Figure 3:**
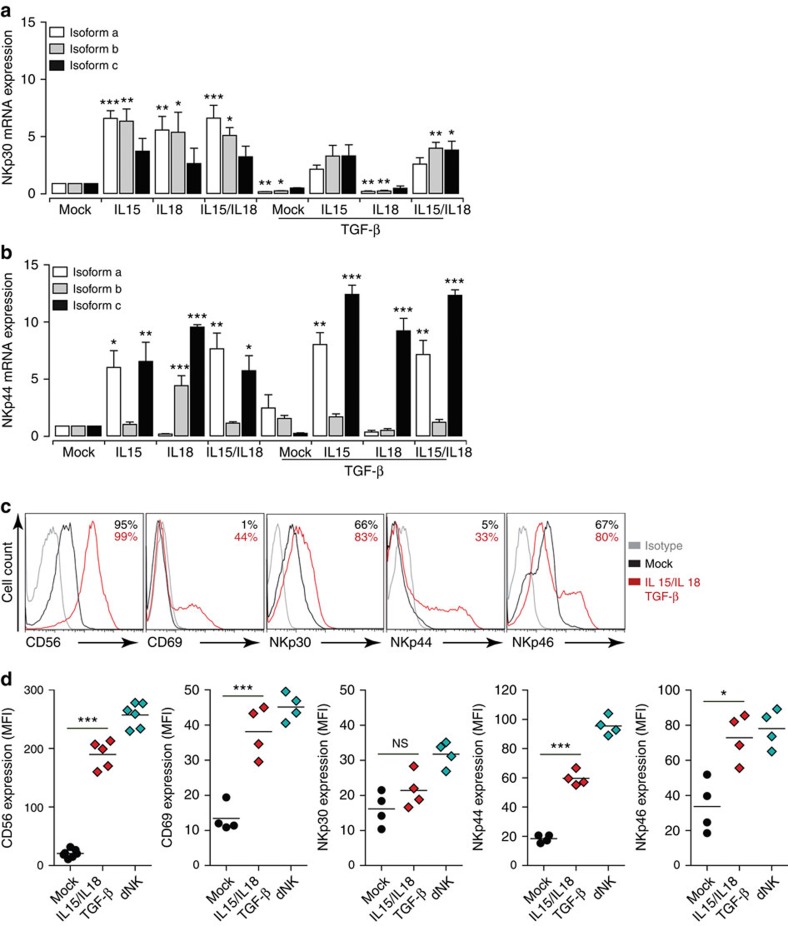
Decidua-enriched cytokines affect the expression of NCR isoforms and modulate pNK cell phenotype. (**a**,**b**) mRNA was extracted from pNK cells that has been cultured for 6 days in media supplemented with IL15, IL18 and TGF-β, alone or in different cytokine combinations. The relative expression of the three NKp30/NCR3 (**a**) and NKp44/NCR2 (**b**) splice variants was then determined by quantitative reverse-transcription PCR (qRT–PCR) analysis. The expression of each isoform was compared with that in cells cultured in cytokine-free conditions (mock). Bar graphs represent mean values±s.e.m. from four independent experiments. *P<0.05, **P<0.01, ***P<0.001; NS, not significant; one-way analysis of variance with Bonferroni post test. (**c**,**d**) Modulation of NK cell phenotype by a decidual cytokine cocktail. (**c**) Flow cytometry analysis showing a representative pNK cell phenotype after 6 days of culture with or without the cytokine cocktail. CD3^neg^CD56^pos^ NK cells were analysed for the indicated markers (*x* axis). Isotype matched controls (grey), pNK cells cultured in complete media (continuous black) and pNK cells cultured in media supplemented with the cytokine cocktail (dashed red) are shown. The percentage of positive cells is indicated for each condition. (**d**) Graphs represent MFI of pNK cells after 6 days of culture with or without the cytokine cocktail and freshly isolated dNK cells. Data on graphs represent mean values±s.e.m. from at least four independent donors. *P<0.05, **P<0.01, ***P<0.001; NS, not significant; paired Student's *t*-test.

We therefore analysed the potential role of these cytokines in altering the expression of NCR splice variants in pNK cells. We evaluated the expression of NKp30 and NKp44 transcripts in pNK cells that were cultured for 6 days in the presence of IL15, IL18 and TGF-β combinations. Compared with untreated pNK cells, IL15 induced a significant increase in NKp30a and NKp30b mRNA (Fig. 3a). TGF-β decreased the relative mRNA levels of all three NKp30 transcripts (Fig. 3a). IL15 tempered the TGF-β effect, although this was not significant. Similar to IL15, IL18 alone increased the level of NKp30a and NKp30b mRNA but its presence did not override the inhibitory effect of TGF-β. Compared with untreated or with TGF-β-treated pNK cells, TGF-β/IL15/IL18 significantly increased NKp30b and NKp30c variants (Fig. 3a). The TGF-β/IL15/IL18 cocktail increased NKp30b and NKp30c levels to a greater extent than NKp30a.

We next examined the expression of NKp44 splice variants. The presence of IL15, IL18 and TGF-β alone or in combination induced a significant change in the expression of NKp44a and NKp44c without considerably affecting the expression of NKp44b mRNA (Fig. 3b). IL15 increased NKp44a and NKp44c levels, whereas IL18 downregulated NKp44a but increased NKp44b and NKp44c. IL15/IL18 in the presence or absence of TGF-β induced the highest upregulation of NKp44a mRNA (Fig. 3b). IL18 was more effective at increasing levels of the NKp44c isoform and again IL15/IL18, in the presence or absence of TGF-β, significantly upregulated its expression (Fig. 3b). Compared with NKp44b, TGF-β/IL15/IL18 induced a significant increase in NKp44a and NKp44c isoforms. Thus, an environment enriched in TGF-β, IL15 and IL18 shifts the expression of NKp30 and NKp44 splice variants in pNK cells towards that of dNK cells.

To assess the significance of such a shift, we analysed the expression level of several NKRs (CD56, CD16, CD69, NKp46, NKp44, NKp30, NKG2C and NKG2D). In the presence of TGF-β/IL15/IL18 cocktail, and consistent with a previous report^36^, pNK cells upregulated their CD56 expression and >98% of the cells become CD56^bright^. Cells showed a significant increase in mean fluorescence intensity (MFI), with values of 200.6±9.6 compared with 20.7±2.6 observed for cells cultured in cytokine-free medium; thus, the pNK cells had reached levels comparable to those observed for dNK cells (259±8.4; Fig. 3d). A lower CD56 MFI increase was also observed when pNK cells were cultured with IL15 (117±12) or TGF-β/IL15 (164±4; Supplementary Fig. 3b). The presence of IL15 also significantly increased CD69 expression. CD69, barely detected in freshly isolated pNK cells (Fig. 3a), was expressed by >40% of IL15-, IL15/IL18- or TGF-β/IL15/IL18-cultured pNK cells (MFI of 38.4±5), reaching a level similar to that of dNK cells (MFI of 45±5; Fig. 3c,d and Supplementary Fig. 3b). IL15 alone or in combination with IL18, TGF-β or both also allowed the maintenance of CD16 expression in >70% of cells and none of the cytokine combinations altered the CD16 expression in pNK cells (Supplementary Fig. 3a,b).

More than 60% of pNK cells expressed NKp30 and the presence of IL15 promoted this expression (Supplementary Fig. 3b). TGF-β did not affect the baseline expression of NKp30 but significantly tempered the effect of IL15 or IL15/IL18. The TGF-β/IL15/IL18 combination supported the NKp30 level at a level similar to that of dNK cells (Fig. 3d and Supplementary Fig. 3b). Treatment of pNK cells with IL15 alone or in combination with the other two cytokines induced significant increase of NKp44 expression in pNK cells (Supplementary Fig. 3b). MFI comparisons showed very low variability among the different donors, with TGF-β/IL15/IL18 treatment increasing the expression of NKp44 in pNK cells and shifting them towards a dNK cell phenotype (Fig. 3d). Finally, the presence of IL15 upregulated the level of NKp46 but the addition of TGF-β had no effect (Supplementary Fig. 3b) and nearly 80% of cells expressed NKp46 at a level similar to that of dNK (Fig. 3c,d).

We also tested whether expression of NKG2C and NKG2D is alerted on treatment with cytokines. Only 60% of TGF-β/IL15/IL18-treated cells expressed the NKG2C receptor with an expression level comparable to that of freshly isolated pNK or dNK cells. Treatment with TGF-β/IL15/IL18 slightly increased the percentage of NKG2D-expressing cells and the level of expression to one comparable to that displayed by dNK cells (Supplementary Fig. 3a,b).

Overall, our data attribute an important role to the cytokine-defined microenvironment in shaping up and maintaining the molecular profiles of transcription of the NKp30/NCR3 and NKp44/NCR2 mRNA splice variants in pNK and dNK cells, and their differential expression of the NKRs.

### Cytokine-treated pNK cells acquire dNK cell markers

**Figure 4:**
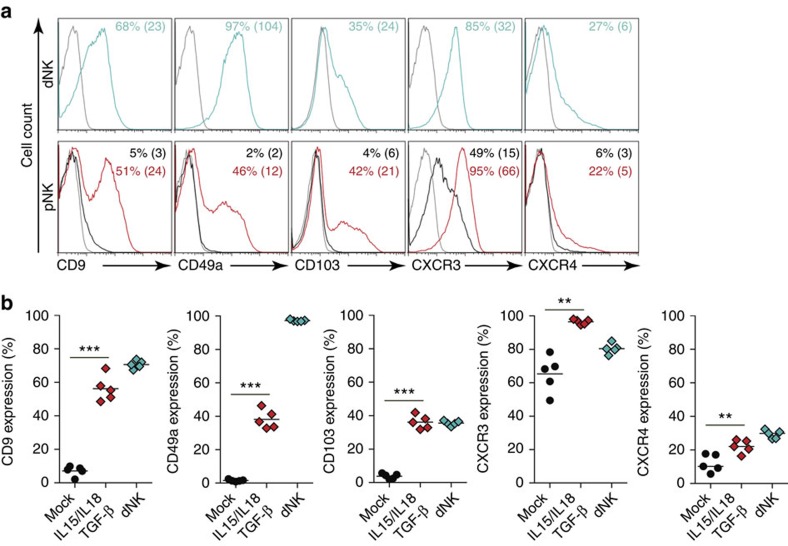
pNK cells acquire specific dNK cell markers on cytokine treatment. The expression of dNK cell-specific markers was analysed by flow cytometry in freshly isolated dNK cells or in pNK cells after 6 days of culture in media supplemented or not with IL15, IL18 and TGF-β. (**a**) Representative FACS profiles of cytokine-cultured pNK cells and freshly isolated dNK cells showing the expression of dNK cell-specific markers (CD9, CD49a and CD103) and two chemokine receptors that are differentially expressed between the two cell subsets (CXCR3 and CXR4). The percentage of positives cells and the MFI are given for each marker. (**b**) Data on graphs represent the percentage of freshly isolated dNK cells and pNK cells cultured for 6 days with or without cytokines. Data are mean value±s.e.m. from at least four independent donors. *P<0.05, **P<0.01, ***P<0.001; NS, not significant; paired Student's *t*-test.

To confirm that TGF-β/IL15/IL18-induced NKp30/NCR3 and NKp44/NCR2 alternative spliced variants polarize pNK cells towards a dNK cell phenotype, we determined the expression of dNK cell-specific markers (CD9, CD49a and CD103)^27^,^36^ and chemokine receptors CXCR3 and CXCR4, which are differentially expressed in tissue-infiltrating lymphocytes^18^,^21^,^37^. pNK cells did not express CD9, CD49a and CD103 or the chemokine receptor CXCR4 (Fig. 4a,b). However, both cell subsets expressed the chemokine receptor CXCR3. IL15, IL18 or a combination of both cytokines did not affect the expression of dNK cell-specific markers or the expression of the chemokine receptors (Supplementary Fig. 4a). The presence of TGF-β alone increased the expression of CD9, CD49a and CD103 in pNK cells and remarkably increased CXCR3 expression while inducing that of CXCR4 receptors (Supplementary Fig. 4a). The presence of IL15 prompted TGF-β-induced expression of CD9, CD49a and CD103, and maintained TGF-β-induced expression of CXCR3 but decreased CXCR4 to the dNK cell levels (Supplementary Fig. 4a). Again, the TGF-β/IL15/IL18 combination induced the most significant increase of dNK-specific markers in pNK cells (Fig. 4 and Supplementary Fig. 4a). TGF-β/IL15/IL18-treated pNK cells also expressed both CXCR3 and CXCR4 at levels similar to those of dNK cells. Thus, similar to their effect on the expression of NKp30 and NKp44 splice variants and the differentially expressed receptors, TGF-β/IL15/IL18 cytokines were able to shape the phenotype of pNK cells and shift them towards a dNK cell-specific phenotype. These major changes were not due to cell proliferation or cell death. In fact, only a slight increase in proliferation was observed after 6 days of culture with IL15 and cell death was negligible (Supplementary Fig. 4b–d).

These findings provide further evidence that the phenotype and probably the effector functions of dNK and pNK cells are controlled at least in part by their cytokine-defined microenvironment.

### Decidua-enriched cytokines affect pNK cell lytic function

**Figure 5:**
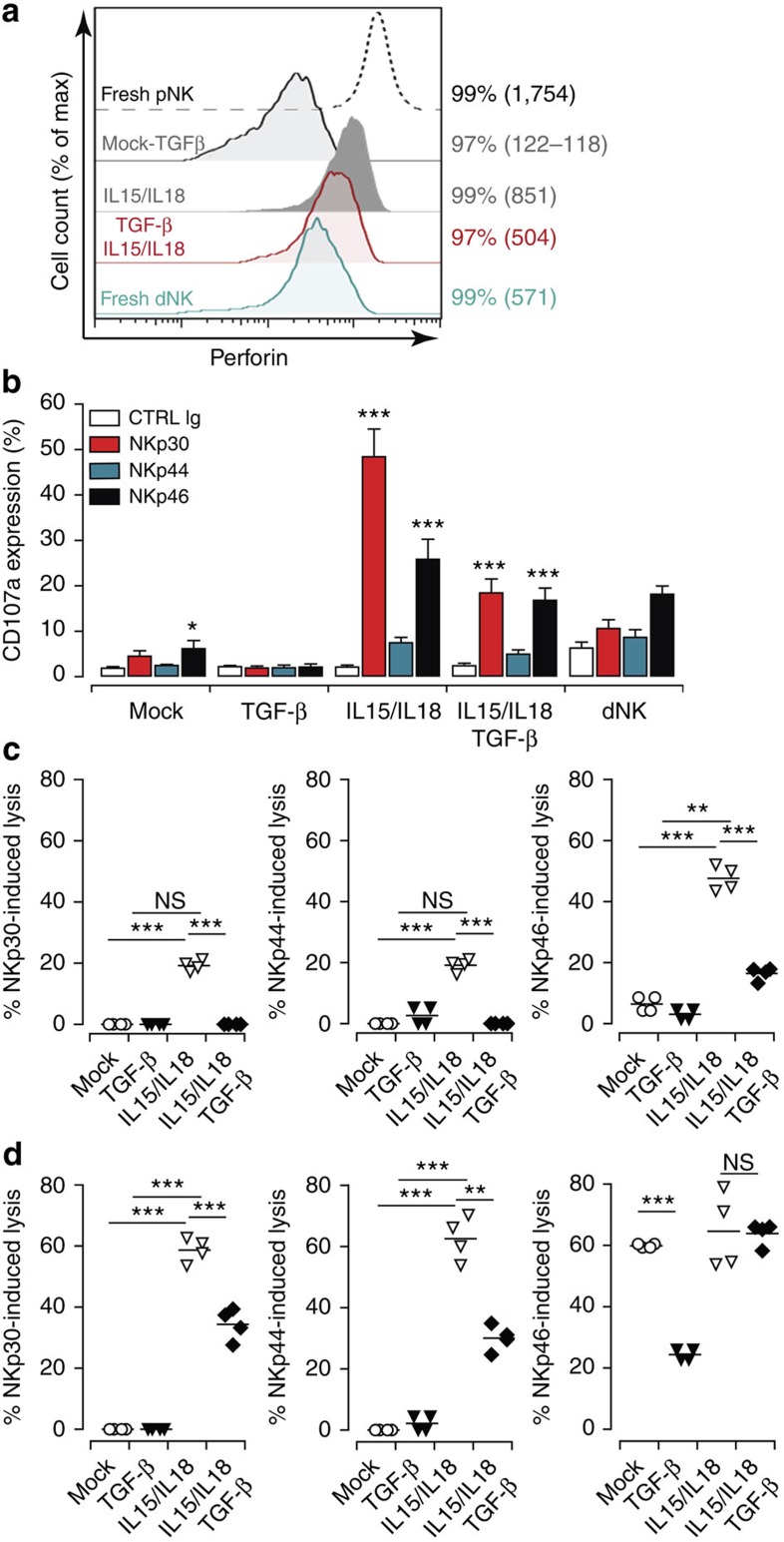
Decidua-enriched cytokines affect pNK cell cytotoxic function. (**a**) The expression of perforin was analysed by flow cytometry in freshly isolated pNK and dNK cells or in pNK cells after 6 days of culture in media alone (Mock) or media supplemented with either TGF-β, IL15/IL18 or the cytokine cocktail (IL15/IL18/TGF-β). The percentage of positives cells and the MFI are given for each marker. (**b**) CD107a cell surface expression in pNK cells cultured for 6 days and freshly isolated dNK cells after 4 h of NCR ligation. NK cells were stimulated with anti-NKp30, -NKp44, -NKp46 antibodies or isotype-matched controls and chilled on ice. Cellular degranulation (CD107a expression) was then assessed by flow cytometry on CD3^neg^CD56^pos^cells. Data on graphs represent mean values±s.e.m. from six independent donors. *P<0.05, **P<0.01, ***P<0.001; NS, not significant; one-way analysis of variance with Bonferroni post test. (**c**,**d**) NK cell cytotoxic activity was evaluated in a redirected lysis assay. ^51^Cr-labelled P815 cells were incubated with mAbs against the indicated receptor or with an IgG control. Cultured pNK cells were used at the effector:target ratio of 1:1 (**c**) or 25:1 (**d**). Data represent mean values±s.e.m. from at least four independent donors. *P<0.05, **P<0.01, ***P<0.001; NS, not significant; one-way analysis of variance with Bonferroni post test.

We next tested whether the cytokine-induced NKp30/NCR3 and NKp44/NCR2 changes affected pNK cell lytic function. First, we tested the effect of cytokines on pNK cell perforin content by flow cytometry. Freshly isolated pNK cells expressed high levels of perforin (Fig. 5a). After 6 days of culture in medium alone or in the presence of TGF-β, even if nearly 100% of pNK cells remained positive for perforin, the expression levels drastically decreased. On treatment with IL15/IL18 or the TGF-β/IL15/IL18 combination, all cells remained positive for perforin but its expression levels significantly decreased (Fig. 5a). The presence of TGF-β/IL15/IL18 cocktail allowed the cells to shift their perforin content to levels similar to that of dNK cells, suggesting that in the presence of a defined cytokine microenvironment pNK cells could also exhibit a dNK-like cytolytic behaviour.

To verify this, pNK cells were cultured with different cytokine combinations and their lytic function was assessed through analysis of CD107a expression after NCR ligation (Fig. 5b and Supplementary Fig. 5a). As expected, pNK cells cultured in cytokine-free medium showed a low degranulation capacity. The IL15-cultured pNK cells significantly degranulated on NKp30, NKp46 and, but to a much lesser extent on, NKp44 ligation (Supplementary Fig. 5a). IL15/IL18 combination showed similar results to IL15 alone (Supplementary Fig. 5a). Treatment with TGF-β did not affect the basal level of CD107a expression in pNK cells. However, the TGF-β/IL15 or TGF-β/IL15/IL18 combinations significantly decreased the capacity of NKp30 and to a much lesser extent NKp44 stimulation to induce CD107a expression. The presence of TGF-β with IL15 or IL15/IL18 slightly reduced the IL15-induced expression of CD107a on engagement of NKp46 but it increased the response induced by NKp46 in untreated pNK cells to a level similar to that of dNK cells (Fig. 5b and Supplementary Fig. 5a).

The co-engagement of NKp30 and NKp46 had no impact on NKp46-induced degranulation of dNK cells, whereas co-engagement of NKp44 and NKp46 significantly affected NKp46-induced degranulation of these cells (Fig. 1c,d). Again, treatment with the TGF-β/IL15/IL18 combination maintained NKp46-induced degranulation lower than IL15/18-maintained pNK cells (Supplementary Fig. 5b). Together, these data indicate that TGF-β/IL15/IL18-treated pNK cells maintain their capacity to degranulate on NCR engagement to a level comparable to that of dNK cells (Fig. 5b).

To provide further support, we assessed the cytotoxic capacity of TGF-β/IL15/IL18-treated pNK cells using P815 target cells. At an effector to target ratio of 1:1, TGF-β- and TGF-β/IL15/IL18-treated pNK cells did not induce any significant killing on engagement of NKp30 and NKp44 (Fig. 5c). IL15/IL18-treated pNK cells induced modest killing (nearly 20% of specific lysis) on engagement of NKp30 and NKp44 but were able to induce up to 50% of specific lysis on engagement of NKp46. TGF-β/IL15/IL18-treated pNK cells also induced a modest killing on engagement of NKp46 (Fig. 5c). Similar killing profiles were observed with an effector to target ratio of 25:1 on ligation of NKp30 and NKp44, indicating that TGF-β- and TGF-β/IL15/IL18-treated pNK cells have lower cytotoxic capacity than untreated pNK cells even at the highest ratio (Fig. 5d). By contrast, the engagement of NKp46 induced similar cytotoxic function in both mock-, IL15/IL18- and TGF-β/IL15/IL18-treated pNK cells.

Together these results indicate that decidua-enriched cytokine-treated pNK cells have a reduced cytotoxic capacity that resembles that of dNK cells and further confirm that TGF-β is a critical decidual cytokine for shaping not only the expression of NCRs splice variants but also the dNK cell lytic function.

### Decidua-enriched cytokines shape pNK cell secretome

**Figure 6:**
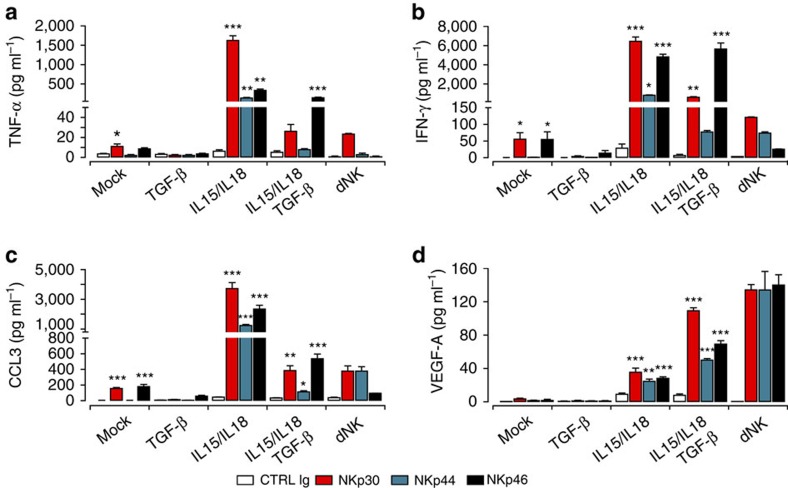
Decidua-enriched cytokines affect pNK cell cytokine secretion. Levels of secreted TNF-α (**a**), IFN-γ (**b**), CCL3 (**c**) and VEGF-A (**d**) by pNK cells cultured for 6 days and freshly isolated dNK cells were measured by multiplexed assay after 18 h NKp30, NKp44 or NKp46 ligation. Cells from five independent donors were pelleted and supernatants were collected. Values represent mean values±s.e.m.; *P<0.05, **P<0.01, ***P<0.001; one-way analysis of variance with Bonferroni post test.

We next compared cytokine secretion by pNK cells that had been maintained under different conditions with that of dNK cells, following NCR ligation using Luminex-multiplexed assay. Medium-maintained pNK cells showed low capacity to produce tumour necrosis factor (TNF)-α, interferon (IFN)-γ, vascular endothelial growth factor (VEGF)-A, chemokine (C–X–C motif) ligand-8 (CXCL8) and CXCL10 (Fig. 6 and Supplementary Figs 6 and 7) but they produced substantial amounts of chemokine (C–C motif) ligand-4 (CCL4) in response to NKp30 or NKp46 ligation (Supplementary Fig. 7). Only minor changes were observed for CCL3 on engagement of the NKp30 or NKp46 receptors (Fig. 6). The presence of IL15 induced a strong increase in TNF-α, IFN-γ, VEGF-A, CCL3, CCL4 and CXCL8 after NCR ligation but did not affect the basal level of CXCL10 secretion (Supplementary Figs 6 and 7). IL18 alone had a minor effect on cytokine production and IL15/IL18 showed levels similar to IL15 treatment alone (Supplementary Figs 6 and 7, and Fig. 6). TGF-β alone showed minor effects. Compared with IL15, TGF-β/IL15 or TGF-β/IL15/IL18 treatment strongly decreased the capacity of NKp30 ligation to induce a high amount of TNF-α, IFN-γ and CCL3 but increased the VEGF-A production (Fig. 6 and Supplementary Fig. 6). Similar to NKp30, NKp44 ligation showed a decrease in TNF-α, IFN-γ, CCL3 and CCL4 levels but did not affect the secretion of VEGF-A (Fig. 6 and Supplementary Figs 6 and 7). The addition of TGFβ did not impair NKp46-induced secretion of TNF-α, IFN-γ or CCL3 in IL15-treated pNK cells (Fig. 6). Compared with IL15 treatment, NKp46 ligation resulted in increased VEGF-A production in TGF-β/IL15- and TGF-β/IL15/IL18-treated cells (Fig. 6). None of the cytokines, alone or in combination, was able to induce the secretion of CXCL10, which is highly secreted by dNK cells on NCR ligation (Supplementary Fig. 7).

Overall, and even though the combination of TGF-β/IL15/IL18 does not fully bias the secretion of inflammatory cytokines by pNK cells, it increases VEGF-A secretion while lowering that of TNF-α and IFN-γ in response to NKp30 or NKp44 ligation. This would suggest that the combination of TGF-β/IL15/IL18 results in pNK cells behaving, at least partially, in a similar way to dNK cells. However, TGF-β/IL15/IL18 treatment leads to only minor changes on NKp46 ligation.

## Discussion

*NCR* genes can be transcribed into at least three splice variants^4^,^9^,^10^ but the physiological relevance of the different isoforms is yet to be fully depicted. Nevertheless, the NKp44 receptor has an inhibitory function in the plasmacytoid dendritic cells from tonsils, which has been linked to the presence of an intra-cytoplasmic ITIM motif^11^,^38^, whereas the NKp30c isoform exerts an immunosuppressive function in cancer^10^. Our data reveal that the expression of different NCR splice variants by NK cells delineates functionally distinct subsets and is governed by the cytokine-defined microenvironment. In the context of the dNK cell subset, the differential expression of the NKp30/NCR3 and NKp44/NCR2 isoforms might give them the capacity to contribute to angiogenesis and to trophoblast invasion throughout pregnancy, which to our knowledge, is the first evidence assigning a potential physiological significance to NCR splice variants.

We found that dNK and pNK cell subsets display differential expression patterns for the NKp30/NCR3 and NKp44/NCR2 splice variants. Quantitative RT–PCR analyses revealed a bias of NKp30 and NKp44 isoform expression towards an inhibitory profile in dNK cells compared with pNK cells. This incongruous isoform expression can result in contrasting effector functions following NCR ligation. Although pNK cells are potent cytotoxic effectors and secrete large amounts of pro-inflammatory cytokines, dNK cells are poorly cytotoxic against conventional K562 target cells^31^. In contrast, dNK cells secrete high amounts of soluble factors including cytokines/chemokines and growth factors that are necessary to promote trophoblast invasion and guarantee the correct remodelling of maternal spiral arteries^13^,^14^. In support of this, our data demonstrate that NKp30 and NKp44 ligation are unable to trigger proper MTOC relocalization, lytic granule polarization and degranulation in dNK cells, whereas NKp46 ligation has similar effects on both pNK and dNK cells.

These results are in line with our biochemical studies demonstrating differences in NKp30- and NKp44-activated signalling pathways between dNK and pNK cells, whereas no major differences were observed for NKp46-triggered signalling. Although the complete molecular mechanism remain to be further clarified, differences in the activation of signalling pathways can result in the differential recruitment of adaptor proteins or in the recruitment of different adaptors as has been shown for other NKRs^39^. This notion is supported by pioneering work on NKp30, which demonstrated a differential recruitment of the ITAM-bearing adaptor molecule CD3ζ by different isoforms^10^. Differences in phosphorylation and activation of signalling pathways downstream of these receptors might be part of a negative feedback loop to control receptor-induced activation. Such a scenario could apply to the NKp44 receptor, as two of its isoforms contain ITIM motifs that transmit strong inhibitory signals, which can counteract activating signals emanating from the NKp46 receptor.

Immune cells in the uterine mucosa contribute a great deal to the outcome of pregnancy^26^. dNK cell behaviour is tightly regulated through different pathways, including local activating/inhibitory signals provided by the fetal trophoblast, cytokine production by the mucosal stroma and autocrine inhibition via the dNK cell's own production of cytokines. In this study, we reveal that a TGF-β/IL15/IL18 cocktail is sufficient to increase the expression of the NKp30c and NKp44c inhibitory isoforms, and to convey a decidual-like phenotype to pNK cells that is marked by increased levels of CD56 and the *de novo* acquisition of the dNK cell-specific markers CD9, CD49a and CD103, as well as CD69 and the NKp44 receptor. Our results not only demonstrate the key role of soluble factors in controlling the expression of NCR splice variants in NK cells but also underscore the fact that soluble factors present within the decidua could alter the alternative splicing of NCRs, to promote a state of tolerance.

Unlike dNK cells that are widely CD16^neg^, we found that CD16 expression was not impaired in TGF-β/IL15/IL18-maintained pNK cells. In addition, the TGF-β/IL15/IL18 cocktail did not impair NK cell cytotoxicity after NKp46 ligation, yet it downmodulated NKp30- and NKp44-induced degranulation. Furthermore, it biased cytokine-induced secretion towards a decidual profile with decreased TNF-α and IFN-γ production, and the induction of VEGF-A secretion. However, it must be noted that we cannot exclude the involvement of other factors that could also promote fetal tolerance. For example, immunosuppressive factors such as the indoleamine 2,3-dioxygenase, IL10 or hypoxia^40^,^41^,^42^ also ensure the maintenance of a decidual phenotype and promote fetal tolerance. This study did not tackle this aspect, but it has been shown that dNK cells establish a sustained contact with semi-allogeneic fetal-derived trophoblasts that express only NKp30 and NKp44 ligands even if they express only low amounts of classical major histocompatibility complex-I molecules^14^. It is likely to be that sustained engagement of the NKp30 and NKp44 receptors will not only stimulate dNK cell soluble factor's secretion that is essential for a healthy pregnancy but also prevent cytotoxicity. Thus, NKp30 and NKp44 are probably key receptors orchestrating the maintenance of the dNK phenotype.

NCRs strongly activate NK cell cytotoxicity against cancer and various pathogens including HIV-1, influenza, hepatitis viruses and *Plasmodium falciparum*^43^,^44^,^45^. Viruses and cancers have great capacities for hijacking their local environment and escaping NCR pressure^46^,^47^,^48^,^49^. Sophisticated immune evasion mechanisms are usually associated with the loss of NCR function and dampening of cell surface major histocompatibility complex-I. However, it is unclear whether changes in the local environment could regulate the alternative splicing of NCRs. Our data clearly demonstrate that modification of the cytokine milieu can favour the expression of inhibitory rather than activating isoforms and probably dampen NK cell responsiveness. Hence, a default/exacerbation of NK cell effector functions could very well be governed by the local microenvironment to change the balance of dominant receptor isoform expression^50^. In such scenario, restoring an appropriate NCR isoforms ratio might re-establish NK cell effector functions.

Differences in the pNK and dNK cell immune responses are well recognized. Even though such discrepancies are probably due to the engagement of the different receptors or differential expression of the same receptor as in cancer patients^10^, little knowledge, if any, has been gained within a healthy setting. In the present study, and in accordance with our published report^13^, we show that the same NCR triggers different functions in dNK and pNK cell populations through the differential expression of receptor isoforms.

Pregnancy disorders are often associated with defaults in artery remodelling that can be attributed to defective dNK cells. Microenvironment bias with a shift from pro-Th2 cytokines towards pro-Th1 cytokines has been implicated in some of these disorders^51^. For example, alterations of IL15, IL18 and TGF-β levels have been reported in patients with pre-eclampsia, a common pregnancy disorder^52^,^53^,^54^. It is therefore tempting to speculate that alterations in the levels of immunosuppressive and/or immunostimulatory cytokines could influence NCR transcript ratios and participate in the aetiology of the disorder.

Besides providing the first evidence assigning a potential biological/physiological relevance to NCR splice variants, our results propose a new model of the regulation of NK cell functions based on the expression of the NKp30/NCR3 and NKp44/NCR2 isoforms. We also provide evidence of the relevance of cytokines present in the pregnant uterus as a trigger for NK cell responsiveness through the regulation of alternative splicing of the NCRs. Thus, the specific adaptation of NKR expression dictates the appropriate responses, in the same manner to that the role of the local microenvironment is proposed to play a role in generating NK cell memory^55^,^56^. Overall, this study demonstrates the molecular differences of NK cell subsets and defines a unique perspective that cytokines enriched in the mucosal microenvironment maintain the decidual phenotype of dNK cells. Whether this would apply to other immune cells remains an open question. Beyond the still lacking knowledge concerning the expression of natural NKp46 ligand, the fact that semi-allogeneic trophoblasts express only NKp30 and NKp44 ligands reinforces the above notion.

## Methods

### Ethics statement

The study has been approved by the local Research Ethical Committee and the National Biomedicine Agency (PFS08-022). All patients gave their signed informed consents according to the Declaration of Helsinki before sample collection.

### Cell purification

First-trimester decidua basalis (at 8–12 weeks of pregnancy) and blood-matched samples were obtained from healthy women undergoing vaginal elective termination of pregnancy (a cohort of ten donors and age range 18–30 years) as previously described^13^,^16^. Briefly, decidua basalis was isolated from elective pregnancy termination samples (8–12 week of pregnancy), minced and digested with collagenase IV (Sigma-Aldrich, France) for 60 min at 37 °C under gentle stirring. The resulting cellular mixture was filtered, separated over Ficoll-Hypaque density gradient (Amersham Biotech). Cells were washed and allowed to adhere to tissue culture plate overnight at 37 °C in tissue culture medium (RPMI 1640/GlutaMAX, Gibco) supplemented with 10% FCS. dNK cells were purified from the non-adherent cell fraction using MACS negative selection kits (Miltenyi Biotec, France). Usually, dNK cells represent 50% of the non-adherent cell fraction but this number varies with the quality and the size of the tissue (2–8 × 10^6^ dNK cells per decidua). pNK cells were isolated from paired peripheral blood samples (pregnancy samples) or healthy volunteer blood donors. More than 98% of purified cells were CD3^neg^CD56^pos^. In some experiments, NKp44 cell surface expression on freshly isolated pNK cells was induced by overnight culture in the presence of IL15 (10 ng ml^−1^).

### Quantitative RT–PCR

Total cellular RNA was isolated from dNK or pNK cells using an RNeasy kit (Qiagen, France). First-strand complementary DNA was synthesized from 1 μg total RNA using SuperScript III reverse transcriptase and random primers, according to the manufacturer's protocol (Life Technologies, France). PCR primers for *NCR3* (ref. 10), *NCR2* and the *β-actin* housekeeping transcripts were designed using an NCBI primer blast and their positions are indicated in Supplementary Fig. 1a and Table 1. The quantitative RT–PCR data were determined and normalized to the *actin* housekeeping gene according to the standard 2^−ΔCt^ method.

### Antibodies and reagents

Fluorochrome-conjugated anti-human monoclonal antibodies were used. Anti-CD56 (allophycocyanin, 1:50 dilution, BD Pharmingen), anti-CD3 (phycoerythrin-Cy7, 1:100 dilution, BD Pharmingen), anti-CD16 (phycoerythrin, 1:100 dilution, BD Pharmingen), anti-CD69 (fluorescein isothiocyanate, 1:50 dilution, BD Pharmingen), anti-NKG2D/CD314 (phycoerythrin, 1:50 dilution, BD Pharmingen), anti-NKp30/CD337 (phycoerythrin, 1:50 dilution, BD Pharmingen), anti-NKp44/CD336) (phycoerythrin, 1:50 dilution, BD Pharmingen), anti-NKp46/CD335 (phycoerythrin, 1:50 dilution, BD Pharmingen), anti-CD9 (phycoerythrin, 1:50 dilution BD Pharmingen), anti-CD49a (phycoerythrin, 1:50 dilution, BD Pharmingen), anti-CD103 (fluorescein isothiocyanate, 1:50 dilution, BD Pharmingen), anti-CD107a (phycoerythrin, 1:50 dilution, BD Pharmingen), anti-NKG2C/CD159c (phycoerythrin, 1:50 dilution, R&D Systems), anti-CXCR3 (PerCP/Cy5.5, 1:50 dilution, Biolegend), anti-CXCR4 (phycoerythrin, 1:50 dilution, Biolegend), anti-human perforin (phycoerythrin, 1:50 dilution, eBioscience) and isotype-matched controls (1:100 dilution, BD Pharmingen). Recombinant human IL15, IL18 and TGF-β (Tebu-bio, France) were used at a final concentration of 10 ng ml^−1^ for IL15 and IL18, and 2.5 ng ml^−1^ for TGF-β. Phorbol myristate acetate, a protein kinase C activator, and ionomycin, a Ca^2+^ ionophore, were obtained from Sigma-Aldrich and used at a final concentration of 2 ng ml^−1^ and 1 μg ml^−1^, respectively.

### Immunostaining and cytometry analysis

After stimulation, cells were washed in PBS and immunostaining was carried out at 4 °C using fluochrome-coupled monoclonal antibodies diluted in FACS buffer (PBS, 1% BSA). Intracellular staining was performed on fixed cells using the BD Cytofix/Cytoperm buffer (BD Bioscience, France). Analyses were performed on a BD FACS Calibur cytometer with the acquisition of at least 5 × 10^4^ events. Data were further analysed using FlowJo software 7.6.5. FACS plots were obtained by applying a gate on CD3^neg^CD56^pos^ cells. Expression of a given marker is presented as the percentage of positive cells and as the geometric MFI.

### Degranulation assay

For degranulation assay, NK cells were stimulated through receptor ligation using antibody-coated tissue culture plates (10 μg ml^−1^). After 4 h, the reaction was stopped by reducing the temperature to 4 °C. Cells were stained with fluorochrome-conjugated anti-human CD107a or isotype-matched control, then analysed by flow cytometry.

### Confocal microscopy

NK cells were stimulated for 20 min on anti-NKp30-, anti-NKp44-, anti-NKp46- or anti-NKG2A-coated glass coverslips. Cells were then paraformaldehyde fixed and stained with anti-perforin and anti-tubulin antibodies as previously described^13^,^16^. Briefly, filamentous actin cytoskeletons were visualized with AlexaFluor-conjugated phalloidin and nuclei were stained with 4,6-diamidino-2-phenylindole. ISs were analysed using a Zeiss LSM710 confocal microscope (Carl Zeiss, Germany). Images were processed using ImageJ software.

### Redirected killing assay

P815 mastocytoma (ATCC, TIB-64) were used as NK cell targets at an effector to target ratio of 1:1 and 25:1. Target cells (5 × 10^3^ cells) were labelled with 100 μCi of ^51^Cr (sodium chromate, 1 mCi ml^−1^; Perkin Elmer, Courtaboeuf, France) as described^16^. Briefly, ^51^Cr-labelled target cells were incubated for 20 min on ice with specific monoclonal antibodies or isotype-matched controls at a final concentration of 10 μg ml^−1^, then included as targets in NK-cell cytotoxicity assay. After 4 h of co-culture, radioactivity was quantified in cell-free supernatants using Lumaplate and a TopCount (Perkin Elmer). Specific cytotoxicity was calculated as a percentage using the formula (100 × [Sample mean (cpm)−spontaneous mean (cpm)/(maximum mean (cpm)−spontaneous mean (cpm)]). Spontaneous release was obtained from ^51^Cr-labelled target cells cultured alone. Maximum lysis was obtained from cells cultured in 1% Triton X-100.

### NK-cell phenotype

pNK cells were cultured in the presence of the indicated cytokines (2.5 ng ml^−1^ of TGF-β and 10 ng ml^−1^ of IL15 or IL18) for 6 days. Cutlure media were replaced every 72 h and fresh cytokines were added. Cells were immunostained with fluorochrome-conjugated monoclonal antibodies.

### Multiplex cytokine and chemokine array

pNK cells were cultured in the presence of the indicated cytokines (2.5 ng ml^−1^ of TGF-β and 10 ng ml^−1^ of IL15 or IL18) for 6 days. Cultured pNK or freshly isolated dNK cells were stimulated through NCR ligation for 18 h. All experiments were performed using the same number of cells plated overnight. Cytokine, chemokine and growth factor levels were measured in culture supernatants using 7-multiplexed Affymetrix cytokine assay (TNF-α, IFN-γ, VEGF-A, CXCL8/IL-8, CCL3/MIP-1α, CCL4/MIP-1β and CXCL10/IP-10) according to the manufacturer's procedures (Procarta/eBioscience, France).

### Statistical analysis

Statistical analyses were performed using Student's *t*-test or Mann–Whitney test for non-paired groups, paired Student's *t*-test for paired groups and one-way analysis of variance with Bonferroni post test for multiple comparison (GraphPadPrism Software). Data are expressed as mean value±s.e.m. *P*-values<0.05 were considered significant. *P<0.05, **P<0.01, ***P<0.001.

## Additional information

**How to cite this article:** Siewiera, J. *et al.* Natural cytotoxicity receptor splice variants orchestrate the distinct functions of human natural killer cell subtypes. *Nat. Commun.* 6:10183 doi: 10.1038/ncomms10183 (2015).

## Supplementary Material

Supplementary Information Supplementary Figures 1-7 and Supplementary Methods

## Figures and Tables

**Table 1:** Sequences of forward and reverse primers used for PCR analyses.

**Name**		**Sequence**
Actin	Forward	5′-CAAACATGATCTGGGTCATCTTCTC-3′
Actin	Reverse	5′-GCTCGTCGTTCGACAACGGCT-3′
NKp30/NCR3	Forward	5′-TTTCCTCCATGACCACCAGG-3′
NKp30/NCR3 (a)	Reverse	5′-GGACCTTTCCAGGTCAGACATT-3′
NKp30/NCR3 (b)	Reverse	5′-CGGAGAGAGTAGATTTGGCATATT-3′
NKp30/NCR3 (c)	Reverse	5′-TTCCCATGTGACAGTGGCATT-3′
NKp44/NCR2 (a)	Forward	5′-AAGCCCCTGAGTCTCCATCT-3′
NKp44/NCR2 (a)	Reverse	5′-GTTTTCCACCATATGTCCCCC-3′
NKp44/NCR2 (b)	Forward	5′-TTCACAGACCCAGACCCAGAG-3′
NKp44/NCR2 (b)	Reverse	5′-AGGACGGGTGTGAAGGGACA-3′
NKp44/NCR2 (c)	Forward	5′-GTCCCTTCACAGCCACAGAA-3′
NKp44/NCR2 (c)	Reverse	5′-GAGACCTCCCTTGATGCTGC-3′

Primers were designed using NCBI Blast and purchased from Sigma (Sigma, France).
