# Supplementary material for: Natural cytotoxicity receptor splice variants orchestrate the distinct functions of human natural killer cell subtypes
Source: Nat Commun. 2015 Dec 15;6:10183. doi: 10.1038/ncomms10183 (PMC4682172; doi:10.1038/ncomms10183)
Supplement: Supplementary Information — Supplementary Figures 1-7 and Supplementary Methods [file ncomms10183-s1.pdf]

Supplementary Figures

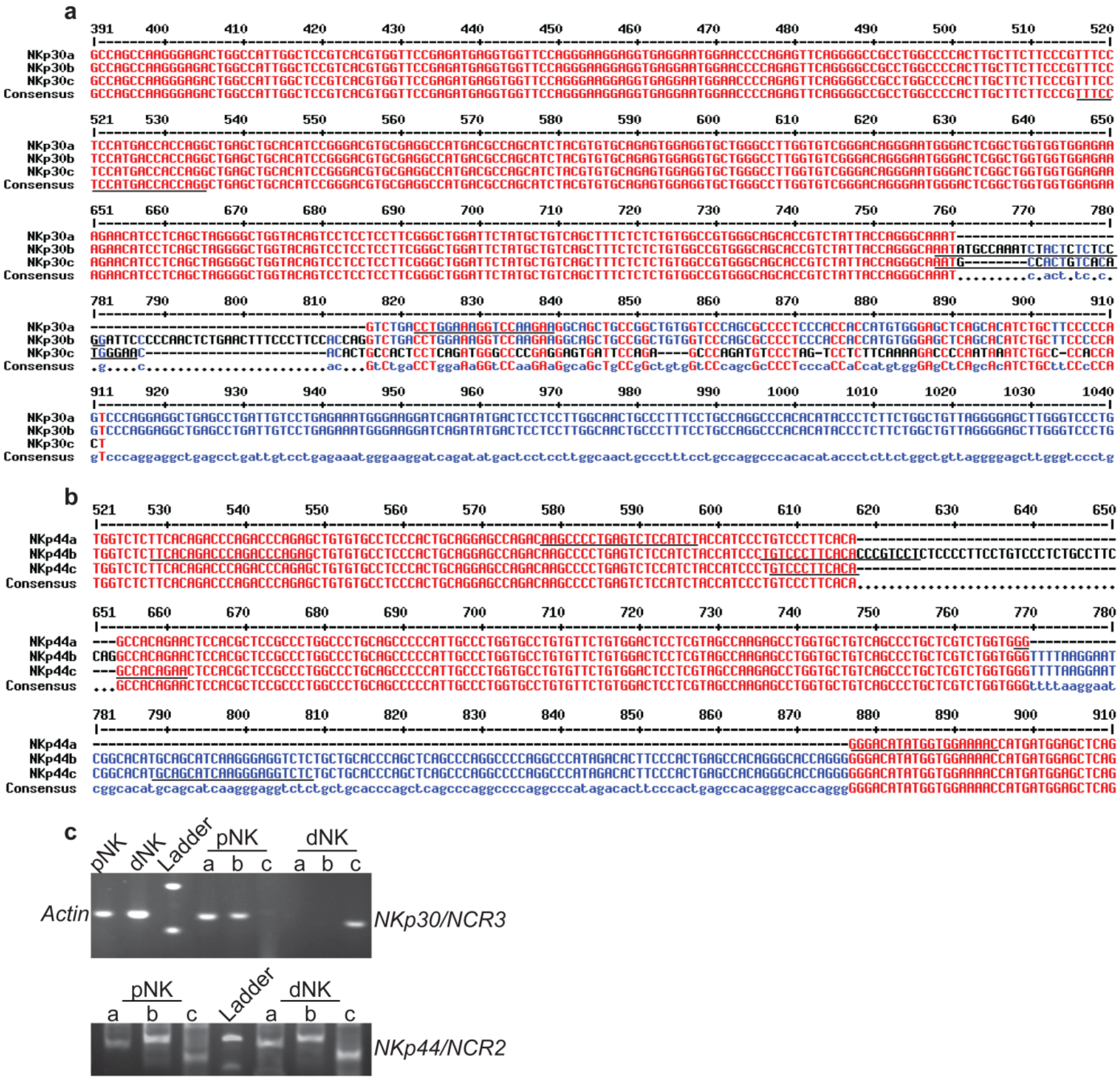

Supplementary Figure 1.

Sequence alignments of NCR splice variants. Multiple alignments of NCR splice variant coding sequence of the (**a**) NKp30/NCR3 splice variant a (NM_147130.2), splice variant b (NM_001145466.1) and splice variant c (NM_001145467.1), and the (**b**) NKp44/NCR2 splice variant a (NM_001199510), splice variant b (NM_001199509.1) and splice variant c (NM_004828.3). Conserved residues are depicted in red. Black indicates insertion and or deletions that are specific for a single splice variant. Blue indicates insertions that are common to two splice variants. Deduced consensus sequence are shown in blue lower-case lettering. Forward and reverse primer sequences are underlined with solid horizontal lines. Numbers above each sequence sets indicate nucleotide position on full length mRNA. Residues conserved in all the aligned sequences are represented by asterisks, while partially conserved residues are marked in blue letters. (**c**) mRNA were extracted from freshly-isolated dNK and pNK cells from the same donor. mRNA expression of the *actin* housekeeping gene, *NKp30/NCR3* and NKp44/NCR2 splice variants was determined by RT-PCR analysis. Actin and NKp30/NCR3 PCR fragments were separated by electrophoresis using a 2% agarose gel. NKp30/NCR3 PCR fragments were separated using a 8% native polyacrylamide gel.

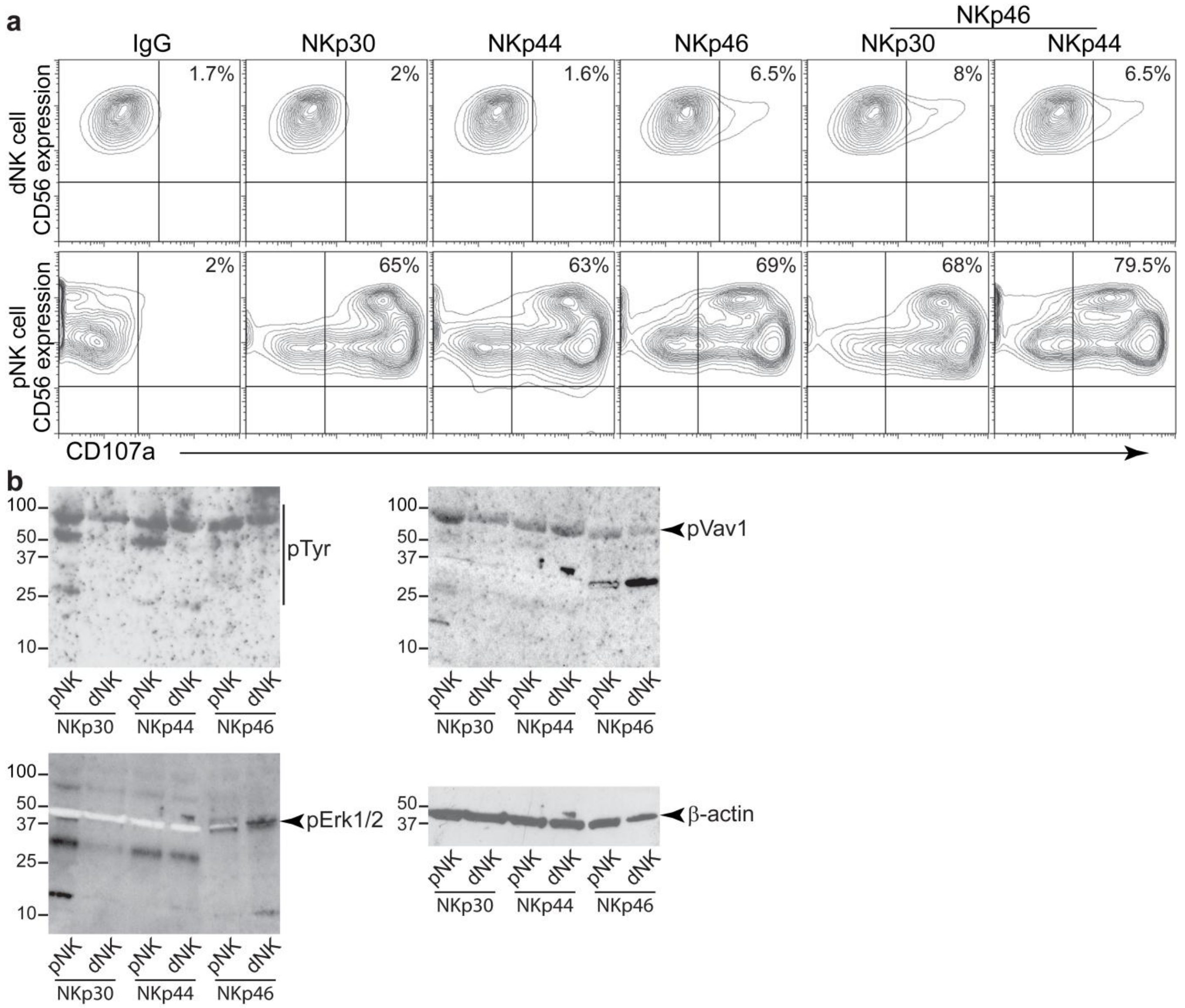

Supplementary Figure 2.

Differential expression of NCR isoforms by dNK cells and pNK cells results in differential activation upon receptor cross-linking. (**a**) Freshly-isolated dNK and pNK cells cultured overnight with IL15 (10 ng ml^{-1}) were stimulated for 4 hours with a single or combination of two specific mAbs. All the reactions were carried in the presence of fluorochrome-conjugated anti-CD107a mAb and monensin. CD107a expression was analysed by flow cytometry on $CD3^{neg}CD56^{pos}$ cells. Representative FACS plots from three independent donors are shown. (**b**) Differential effect of specific receptor ligation on cell signalling. dNK or overnight IL15-cultured pNK cells were stimulated for 20min with anti-NKp30, -NKp44 and -NKp46 antibodies. Cleared cell lysates (10 µg of total cellular proteins) were separated by 4–15% denaturing-gradient gel and electrotransferred. Proteins were successively immunoblotted with the indicated Ab or with anti-β-actin Ab to verify protein loading. Black arrow heads indicate the protein of interest.

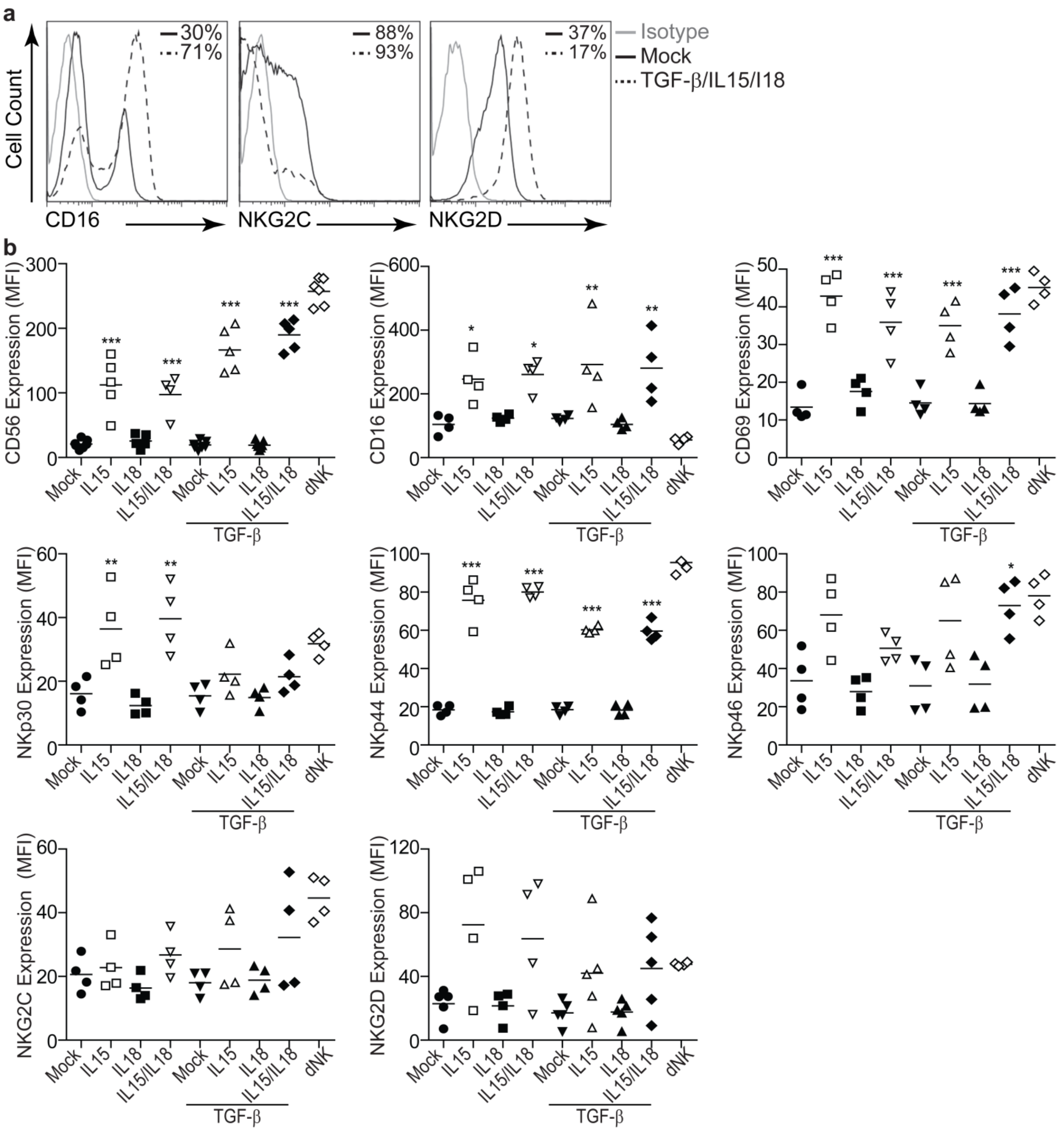

Supplementary Figure 3.

Modulation of NK cell receptor expression by Cytokines enriched in the decidual environment. (**a**) Flow cytometry analysis showing a representative CD3^{neg}CD56^{pos} NK cell expression of CD16, NKG2D or NKG2A after 6 days of culture with different cytokine combinations. Isotype matched control (grey), pNK cells cultured in complete media (continuous black) and pNK cells cultured in media supplemented with cytokine cocktail (dashed black). The percentage of positive cells is given for each condition. (**b**) Graphs represent Mean Fluorescence Intensity (MFI) of pNK cells after 6 days of culture with or without cytokines and freshly-isolated dNK cells. Data on graphs represent mean values ± s.e.m. from at least 4 independent donors. *p<0.05, **p<0.01, ***p<0.001, ns: not significant, one-way analysis of variance with Bonferroni post-test.

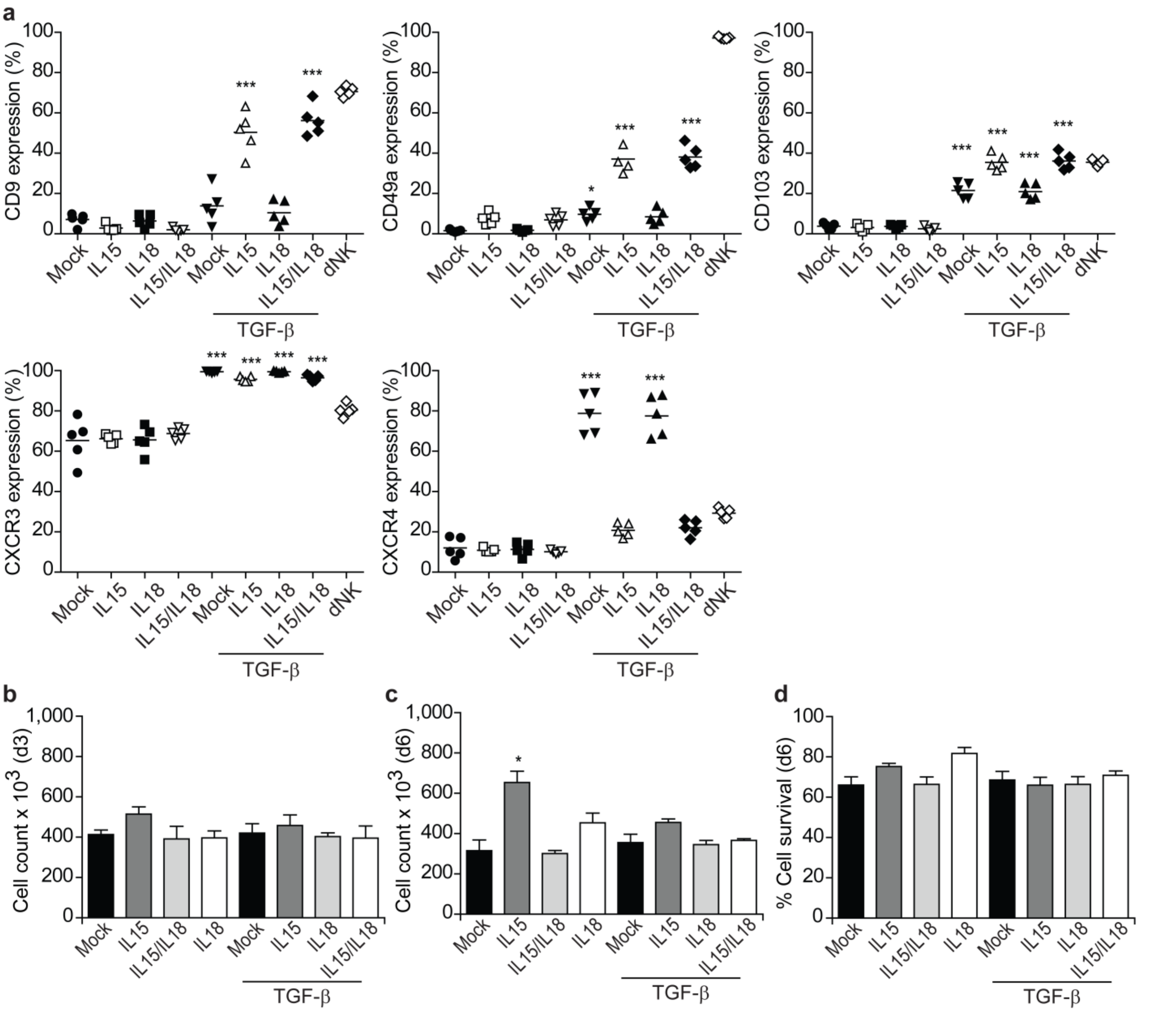

Supplementary Figure 4.

Cytokine treatment induces the acquisition of specific dNK cell markers with no impairment of pNK cell proliferation. The expression of dNK cell specific markers was analysed by flow cytometry in freshly isolated dNK cells and in pNK cells after 6 days of culture in media supplemented or not with different cytokine combinations. Data on graphs represent the percentage of positive cells for a dNK cell specific marker (CD9, CD49a, CD103) and two chemokine receptors that are differentially expressed in the two NK cell subsets (CXCR3 and CXR4). The percentage of positives cells is given for each marker. Data represent mean values ± s.e.m. from at least 4 independent donors. ***p<0.001, one-way analysis of variance with Bonferroni post-test.(**b,c,d**) The impact of cytokines on pNK cell proliferation and survival were monitored by counting cells on hemocytometer before the analysis of PI positive cells by flow cytometry. Cell number after 3 days of culture (**b**), 6 days (**c**), the percentage of cell survival after 6 days of culture (**d**). Each data point represents mean values ± s.e.m. from 4 independent donors performed in triplicates, one-way analysis of variance with Bonferroni post-test.

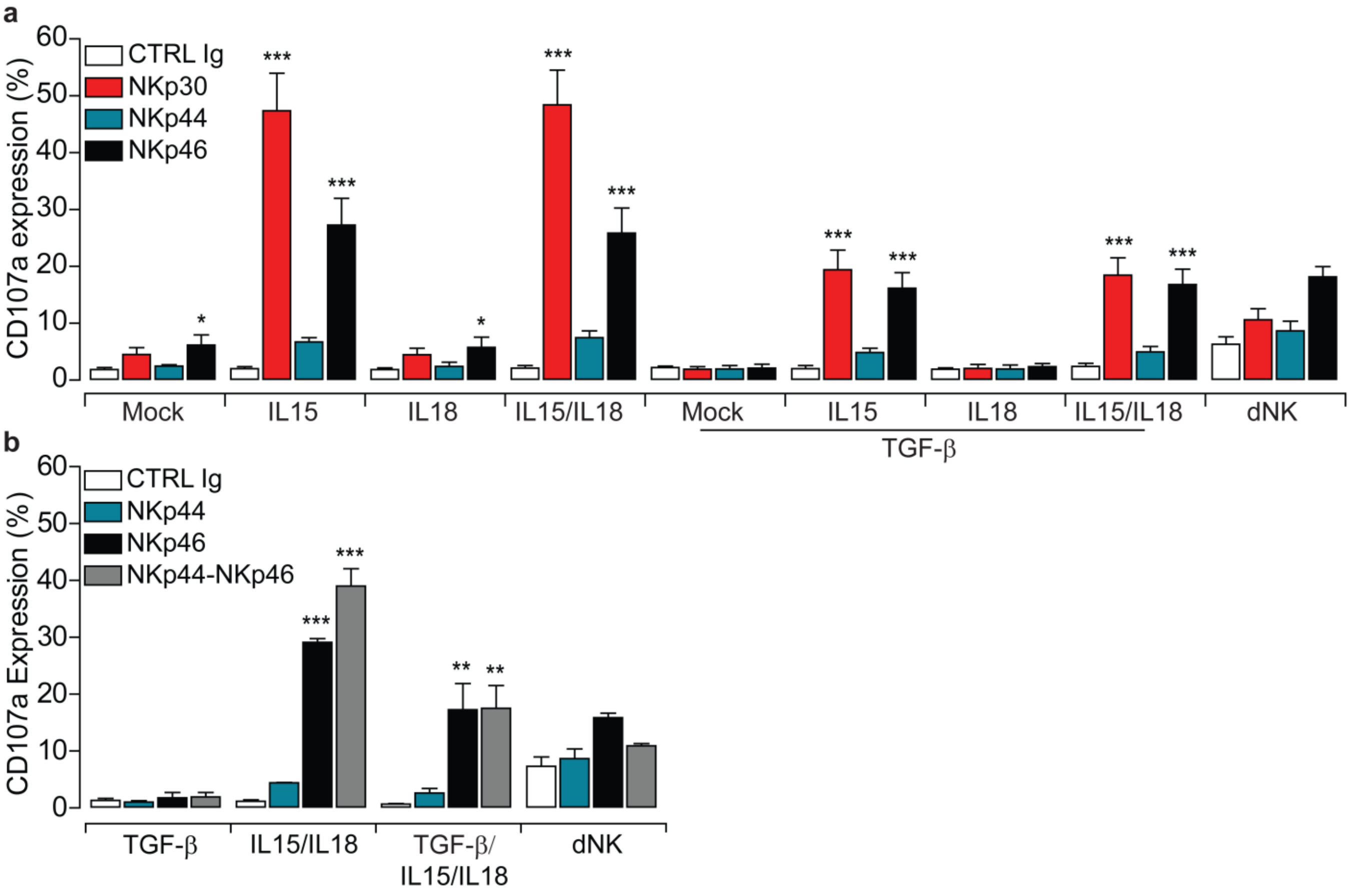

Supplementary Figure 5.

Decidual cytokines affect pNK cell degranualtion. (**a**) CD107a cell surface expression in pNK cells cultured for 6 days and freshly-isolated dNK cells after 4 hours of NCR ligation. NK cells stimulated with anti-NKp30, -NKp44, -NKp46 antibodies or Isotype matched controls and chilled on ice. Cellular degranulation (CD107a expression) was then assessed by flow cytometry on $CD3^{neg}CD56^{pos}$cells. Representative graphs of 6 independent donors are presented. (**b**) Degranulation was evaluated after engagement of NKp46, NKp44 alone or co-engagement of both receptors on cytokine treated cells. Data represent mean values ± s.e.m. from at least 4 independent donors. **p<0.01, ***p<0.001, one-way analysis of variance with Bonferroni post-test.

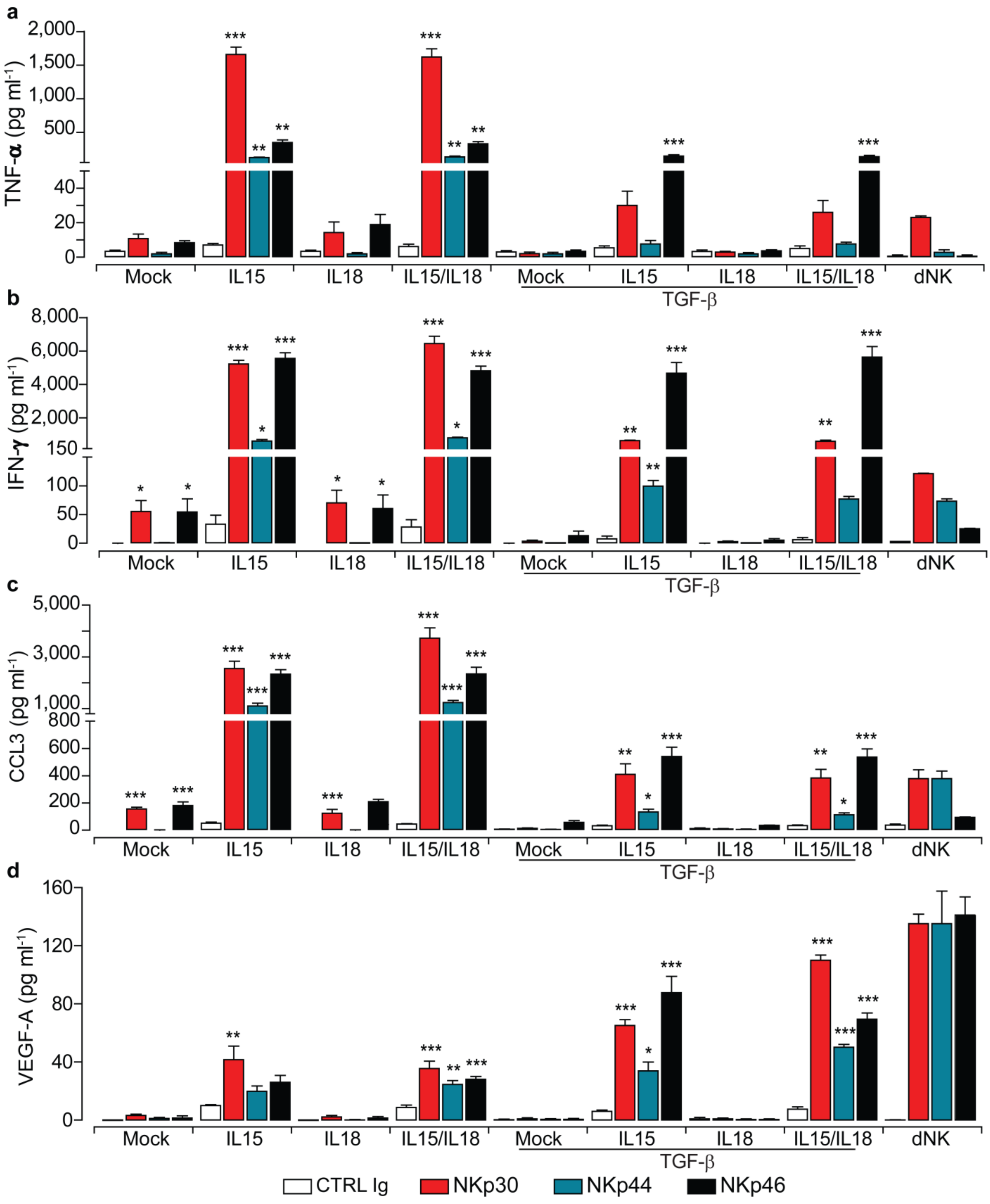

Supplementary Figure 6.

Decidual cytokines affect pNK cell cytokine secretion. Levels of secreted TNF-α (**a**), IFN-γ (**b**), CCL3 (**c**) and VEGF-A (**d**) by pNK cells cultured for 6 days and freshly-isolated dNK cells were measured by multiplexed assay after 18 hours NKp30, NKp44 or NKp46 ligation. Cells from 5 independent donors were pelleted and supernatants were collected. Values represent mean values ± s.e.m. *p<0.05, **p<0.01, ***p<0.001, one-way analysis of variance with Bonferroni post-test.

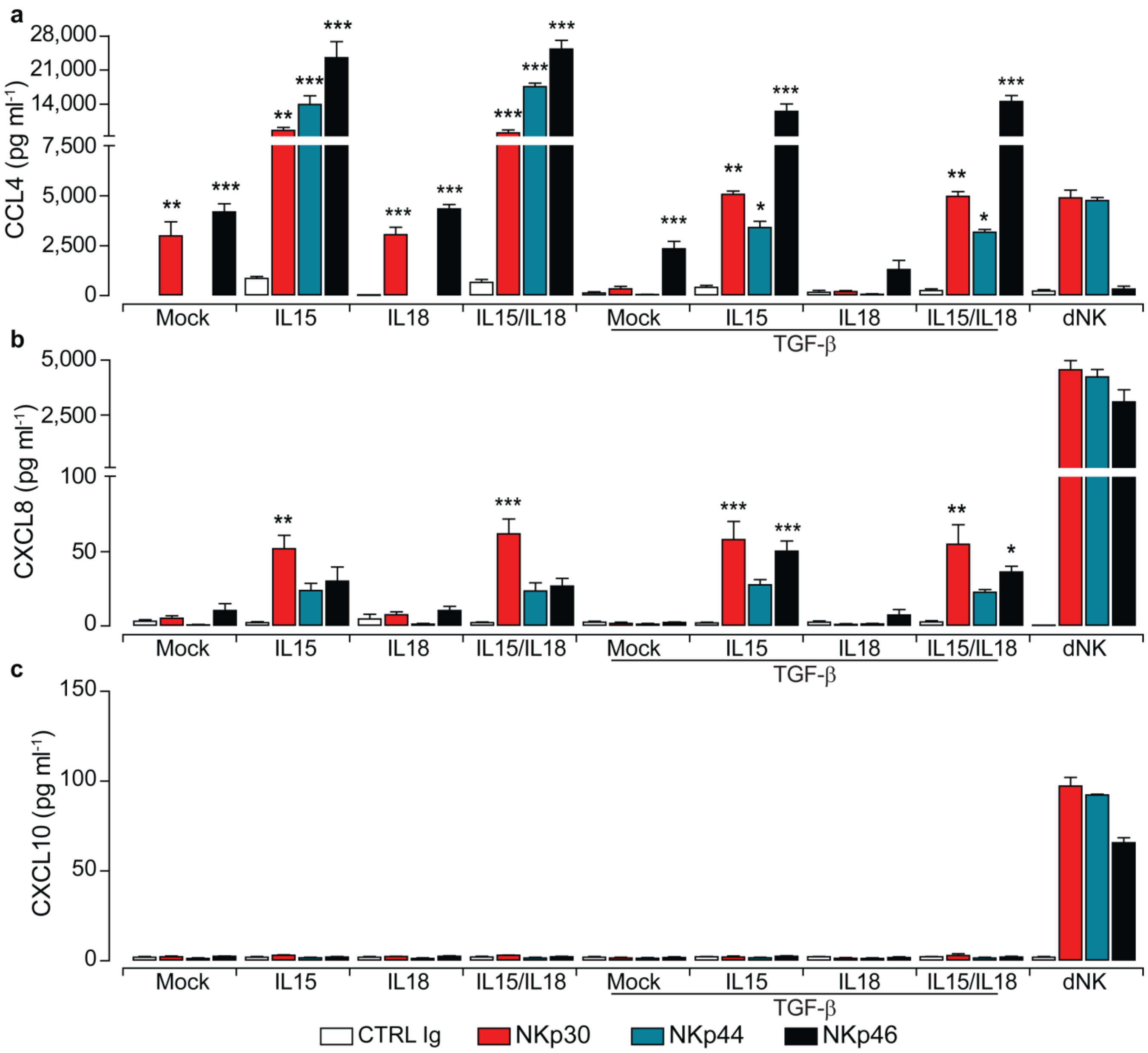

Supplementary Figure 7.

Decidual cytokines affect pNK cell cytokine secretion. Levels of secreted CCL4 (**a**), CXCL8 (**b**), CXCL10 (**c**) by pNK cells cultured for 6 days and freshly-isolated dNK cells were measured by multiplexed assay after 18 hours NKp30, NKp44 or NKp46 ligation. Cells from 5 independent donors were pelleted and supernatants were collected. Values represent mean values ± s.e.m. *p<0.05, **p<0.01, ***p<0.001, one-way analysis of variance with Bonferroni post-test.

Supplementary Methods

RT-PCR

Total cellular RNA was isolated from dNK or pNK cells using an RNeasy kit (Qiagen, France). First-strand cDNA was synthesized from 1 µg total RNA using SuperScript III reverse transcriptase and random primers, according to the manufacturer's protocol (Life Technologies, France). PCR primers for *NCR3*[10], *NCR2* and the *actin* housekeeping transcripts were designed using an NCBI primer blast and their positions are indicated in Supplementary Fig. 1a and Table 1. PCR reactions were performed in a DNA thermal cycler (Perkin-Elmer) as follows: initial incubation at 55°C for 2 min, denaturation at 95°C for 10 min followed by 30 cycles (35 s at 95°C, 45 s at 56°C, 45 s at 72 °C). PCRs were normalized to that of *β-actin* housekeeping gene run for 25 cycles.

Cumulative CD107a staining

To assay cumulative degranulation, CD107a fluorochrome-conjugated antibody (BD-Pharmingen) was added at the beginning of stimulation along with 5 µg ml^{-1} monensin (Sigma Aldrich). After 4 h, cells were chilled at 4°C, washed in PBS and stained for CD3 and CD56 with specific antibodies. Flow Cytometry gates for positive CD107a expression were defined on $CD3^{neg}CD56^{pos}$ unstimulated controls.

Immunoblotting

NK cells were stimulated for 20 min through receptor cross-linking on anti-NKp30- (clone-210847), anti-NKp44- (polyclonal goat IgG) or anti-NKp46-specific antibodies (clone-195314) coated plates. Cells were lysed in sample buffer (1% NP40, 20 mM HEPES (pH 7.9), 10 mM KCl, 1mM EDTA, 1 mM PMSF, 1% glycerol and cocktail of proteases and phosphatases inhibitors). 8 µg of proteins separated 4–15% denaturing-gradient gel, electrotransferred to Immobilon membrane. Immunoblots were performed on the same with anti-phospho tyrosine mouse monoclonal antibody (4G10R-Platinum: Millipore, 1:5,000 dilution), anti-phospho Vav-1 (Tyr 174) rabbit polyclonal antibody (sc-16408-R, Santa Cruz, 1:1,000 dilution), anti-phospho p44/42 MAPK (Erk1/2) (Thr202/Tyr204) rabbit monoclonal antibody (Cell Signaling, 1:5,000 dilution) and normalized to β-actin mouse monoclonal antibody (MAB1501: Millipore, 1:10,000 dilution). After each blotting, the membrane was stripped using mild stripping buffer. Blots were developed with ECL plus (Amersham), according to manufacturer's instructions. Images were captured using BioRad Chemidoc system (BioRad).
